# METABOLOMIC SIGNATURES OF AGE, EXTREME OLD AGE, AND LONGEVITY IN LONG LIFE FAMILY STUDY PARTICIPANTS

**DOI:** 10.1093/geroni/igad104.2057

**Published:** 2023-12-21

**Authors:** Paola Sebastiani, Zeyuan Sog, Michael Lustgarten, Thomas Perls, Stefano Monti, Stacy Andersen, Michaela Schwaiger-Haber, Gary Patti

**Affiliations:** Tufts Medical Center, Boston, Massachusetts, United States; Boston University, Boston, Massachusetts, United States; Tufts University, Boston, Massachusetts, United States; Boston University, Boston, Massachusetts, United States; Boston University, Boston, Massachusetts, United States; Boston University Chobanian & Avedisian School of Medicine, Boston, Massachusetts, United States; Washington University St Louis, St. Louis, Missouri, United States; Washington University St Louis, St. Louis, Missouri, United States

## Abstract

Serum metabolomics of aging has been a growing area of interest and several studies have identified metabolites that correlate with chronological age. The Long Life Family Study (LLFS) has generated serum metabolomics of individuals aged 30 to 110 years and several years of follow up, and offers a unique opportunity to identify (1) a robust signature of age; (2) a signature of extreme old age that differs from the age-related signature; and (3) a signature that predict survival. We analyzed 409 general metabolites and lipid species in approximately 2700 LLFS participants and used a state-of-the-art computational approach with mixed effect models and whole genome sequence data to model within family relation. The analysis identified 305 metabolites that correlate with age at 5% false discovery rate (FDR), 30 metabolites that are not age related and differ in centenarians compared to younger individuals, and 144 metabolites that predict survival at 5%FDR. The aging signature included well known markers: eg ergothioneine and tryptophan that decreased with older age, and was enriched for carbohydrates, organic acids, and several lipid classes. The extreme old age signature was enriched for glycerolipids and glycerophospholipids. The metabolomics signature of survival was enriched for nucleic and organic acids. Comparison with other studies showed strong agreement of results and also highlighted unique finding in extreme old individuals. The analysis also showed substantial variability of serum metabolomics at different ages, thus confirming the heterogeneity of molecular signatures of age and the opportunity to discover specific molecular profiles that promote heathy aging.

